# Engineered miniature H1 promoters with dedicated RNA polymerase II or III activity

**DOI:** 10.1074/jbc.RA120.015386

**Published:** 2020-11-23

**Authors:** Zongliang Gao, Yme Ubeles van der Velden, Minghui Fan, Cynthia Alyssa van der Linden, Monique Vink, Elena Herrera-Carrillo, Ben Berkhout

**Affiliations:** 1 Laboratory of Experimental Virology, Department of Medical Microbiology, Amsterdam UMC, Academic Medical Center, University of Amsterdam, Amsterdam, the Netherlands; 2 Department of Biomedicine, Aarhus University, Aarhus, Denmark

**Keywords:** H1 promoter, RNA polymerase, Pol II, Pol III, engineering, BRF, b-related factor, DSE, distal sequence element, EF1a, elongation factor 1a promoter, FCS, fetal calf serum, HCT116, colon cancer cell lines, HEK293T, human embryonic kidney 293 T cells, PICs, preinitiation complexes, Pol, RNA polymerase, PSE, proximal sequence element, Staf, staf binding site, SV40, simian virus 40, TZM-b1, JC53-bl [clone 13]

## Abstract

RNA polymerase III (Pol III) promoters, such as 7SK, U6, and H1, are widely used for the expression of small noncoding RNAs, including short hairpin RNAs for RNAi experiments and guide RNAs for CRISPR-mediated genome editing. We previously reported dual RNA polymerase activity (Pol II/III) for the human H1 promoter and demonstrated that this promiscuous RNA polymerase use can be exploited for the simultaneous expression of both a noncoding RNA and an mRNA. However, this combination is not a desired feature in other experimental and therapeutic settings. To overcome this limitation of the H1 promoter, we engineered a miniature H1/7SK hybrid promoter with minimal Pol II activity, thereby boosting Pol III activity to a level that is higher than that of either parental promoter. In parallel, we also engineered small Pol II-specific H1 promoter variants and explored their use as general Pol II promoters for protein expression. The newly engineered promoter variants form an attractive alternative to the commonly used H1 promoter in terms of not only activity and small promoter size but also concerning safety by exclusive expression of the desired therapeutic transcript (either pol II or pol III but not both).

Type 3 RNA polymerase (Pol III) promoters, such as 7SK, U6, and H1, are popular for the expression of small noncoding RNAs because of their robust level of transcription in all cell types and defined transcription initiation and termination sites, such that a precise (therapeutic) transcript is made (1, 2). These promoters are unique in that all critical elements are located upstream of the transcriptional initiation site, thus enabling the expression of almost any small RNA sequence, including siRNA or shRNA for RNAi purposes, and guide RNA for CRISPR-mediated genome editing applications (1, 2, 3, 4).

We previously reported that these Pol III promoters can also load Pol II and synthesize lengthy translation-competent mRNAs (5). These three promoters differ profoundly in Pol II strength (H1 >> U6 > 7SK). Pol II strength of H1 is higher than that of the commonly used simian virus 40 (SV40) early Pol II promoter, whereas 7SK exhibits only minimal Pol II activity. We previously estimated a 3-fold ratio of Pol III to Pol II transcription by the H1 promoter (5). Thus, among these three promoters, 7SK is the promoter of choice in terms of Pol III specificity, whereas H1 can hardly be considered a Pol III-specific promoter. This dual activity of the H1 promoter does however provide an ingenious possibility for the simultaneous expression of both a small noncoding RNA and an mRNA. We explored this unique feature to coexpress both a guide RNA and the Cas9 endonuclease, thus constituting a single promoter-driven CRISPR–Cas9 system that provides a significant titer advantage in lentiviral vector production (6). We also envisioned that one could modulate the Pol II/III ratio to further broaden the applicability of the H1 system. In a previous study, we showed that mutation of the TATA box of type 3 RNA Pol III promoters, including H1, abolished Pol III activity and enhanced Pol II activity, the latter presumably caused by the loss of competitive Pol III binding to the promoter (5). The reverse strategy, which is to construct a Pol III-specific H1 promoter, is also of interest as this would not only boost the expression of the small RNA but also—perhaps more importantly—disrupt the generation of undesired and lengthy Pol II transcripts that may even encode an immunogenic protein, a scenario that is to be avoided, especially in clinical gene therapy settings.

The profound difference in Pol II activity of the H1 and 7SK promoters is quite remarkable as they share a similar type 3 RNA Pol III promoter architecture (7). Both promoters consist of a basal region directing basal transcription and a distal sequence element (DSE) that enhances transcription (2) (Fig. 1*A*). The basal region is composed of a proximal sequence element (PSE) and a TATA box that are separated by a spacer. The DSE encodes an octamer motif and a Staf binding site (Staf). However, the relative position and exact nucleotide sequence of these elements differ between H1 and 7SK (7). For example, these elements are distributed over approximately 240 bp of the 7SK promoter, whereas they lie within a compact 100-bp region of the H1 promoter. Based on the overall similarity in promoter architecture, we hypothesized that it would be possible to engineer a Pol III-specific H1 promoter by replacing H1 elements by corresponding 7SK motifs. To this end, we first delineated which sequences of the H1 promoter are critical for Pol II/III activity and specificity. By systematic swapping of elements, we engineered a compact H1/7SK hybrid promoter with enhanced Pol III activity and specificity. In parallel, we also performed an in-depth analysis of two Pol II-specific H1 variants with a mutated TATA box and explored their potential as general Pol II promoters for protein expression.

**Figure 1:**
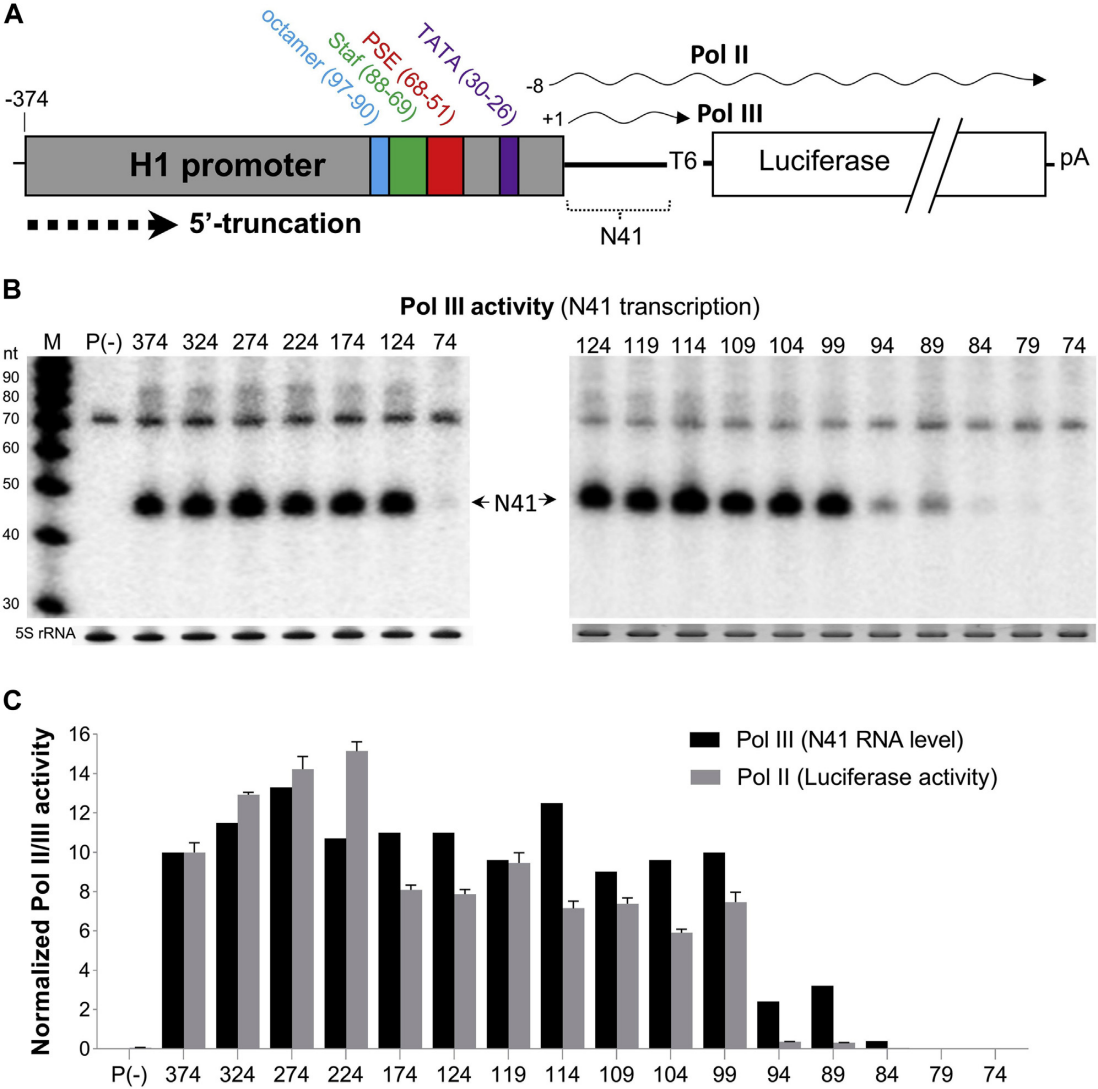
**Truncation analysis of H1 promoter.***A,* schematic of the H1 promoter construct for measuring the Pol II/III activity. The full-length H1 promoter was truncated from the 5'-end by 50-bp steps up to position 74 (all numbers relate to the distance from the transcription start site). Pol III activity drives the expression of a 41-nt small RNA (N41) that starts at +1 position and is terminated at the T6 signal, yielding transcripts with a variable tail of 1 to 6 U residues (21). Pol II produces the luciferase mRNA that initiates at the −8 position and is terminated at the SV40 polyadenylation (pA) signal. *B,* N41 short transcript detection was performed by Northern blotting. Equimolar amounts of DNA constructs were transfected into HEK293T cells, and total cellular RNA was harvested after 2 days post-transfection. The P(−) plasmid that lacks a promoter was used as negative control. An equal amount of total RNA was used for Northern blotting for N41 detection. M, RNA size marker (nt) is shown on the left. Ethidium bromide staining of 5S rRNA (below the panels) served as loading control. The position of the N41 transcript is indicated. Representative blots are shown. *C,* the Pol II/III activity profile of the H1 truncation variants is shown. N41 RNA level (Pol III activity) was normalized to 5S RNA as shown in *panel B*, and the activity measured for H1-374 was arbitrarily set at 10. To measure Pol II activity, we performed luciferase measurements. We transfected equimolar amounts of H1 constructs into HEK293T cells. A fixed amount of Renilla plasmid was cotransfected as control for transfection efficiency. P(−) was used as negative control. The dual-luciferase assay was conducted 2 days after transfection, and the ratio of firefly/renilla was calculated to determine the relative firefly activity. Luciferase activity measured for full-length H1 was arbitrarily set at 10. The data represent the mean ± S.D. (n = 3). Pol II/III, RNA polymerase II/III; PSE, proximal sequence element; Staf, Staf binding site; SV40, simian virus 40.

## Results

### Delineating the H1 promoter elements critical for Pol II/III activity

A previous H1 promoter study showed that the important Pol III transcription elements are located within 100 bp (H1-100) immediately upstream of the transcription initiation site (7). Regardless, the H1 promoter that is generally used for small RNA expression is at least 220-bp long (4, 8). The elements required for Pol III activity of the H1 promoter are known, but whether these elements are also required for the recently discovered Pol II activity remains unknown. To address this, we first delineated the H1 boundaries for Pol II and III activity. The basal region near the 3'-end with the PSE and TATA box is supposed to be essential for transcription. We therefore introduced serial 5' truncations of 50 bp in the full-length 374-bp H1 promoter, producing a set of six promoter variants (Fig. 1*A* and *left panel* of Fig. 1*B*). To measure the promoter activities, the H1 variants were designed to express both a 41-nt small RNA (N41) by Pol III and a 1650-bp luciferase mRNA by Pol II (Fig. 1*A*). Equal molar amounts of the DNA constructs were transfected into human embryonic kidney 293T (HEK293T) cells, and cell extracts were prepared after 2 days. Pol II activity was quantified by luciferase assays, and Pol III activity was assessed by a Northern blot for the N41 transcript.

The short N41 transcript was abundantly expressed by the full-length H1-374 construct, and similar expression levels were observed down to construct H1-124, but expression was lost completely for H1-74 (*left panel* of Fig. 1*B*, quantitation is shown in Fig. 1*C*). This result indicates that the sequence between position −124 and −74 is critical for Pol III activity. To map the critical elements in more detail, we introduced serial 5-bp truncations in H1-124. The constructs with deletions up to H1-99 did fully sustain Pol III transcriptional activity (*right panel* of Fig. 1*B*, quantitation is shown in Fig. 1*C*), but the smaller promoter variants either lost most (H1-94 and H1-87) or nearly all (H1-84, H1-79, and H1-74) Pol III activity. The 5-bp deletion going from H1-99 to H1-94 disrupts the octamer motif at position 97 to 90 (Fig. 1*A*), thus confirming the importance of this DSE enhancer for Pol III transcription. These results are also in line with the report that identified H1-100 as the minimal Pol III promoter (7).

Intriguingly, the Pol II activity profile based on luciferase assays for the set of truncated H1 promoters showed a similar trend as the Pol III activity (Fig. 1*C*). The first four deletion variants remain active, and the Pol II promoter activity is in fact somewhat increased such that H1-224 is 1.5- and 2-fold more active than H1-374 and H1-124, respectively. The Pol II activity drops to a level slightly below that of H1-374 for the subsequent deletions in H1-174 to H1-99. Deletion of the octamer in H1-94 and H1-89 nearly abrogated Pol II transcription, even to a greater extent than the concomitant drop in Pol III transcription. Full Pol II inactivity is scored for H1-84, H1-79, and H1-74. The overall Pol II/III activity profiles are thus quite similar, suggesting that Pol II and III complexes may use the same promoter elements and indicating that the octamer is critical for both activities. In summary, these initial results show that H1-99 (hereafter referred to as the H1-core) contains the minimal sequences required for both Pol II and III activity.

### Engineering of an H1-core promoter with improved Pol III activity and specificity

**Figure 2:**
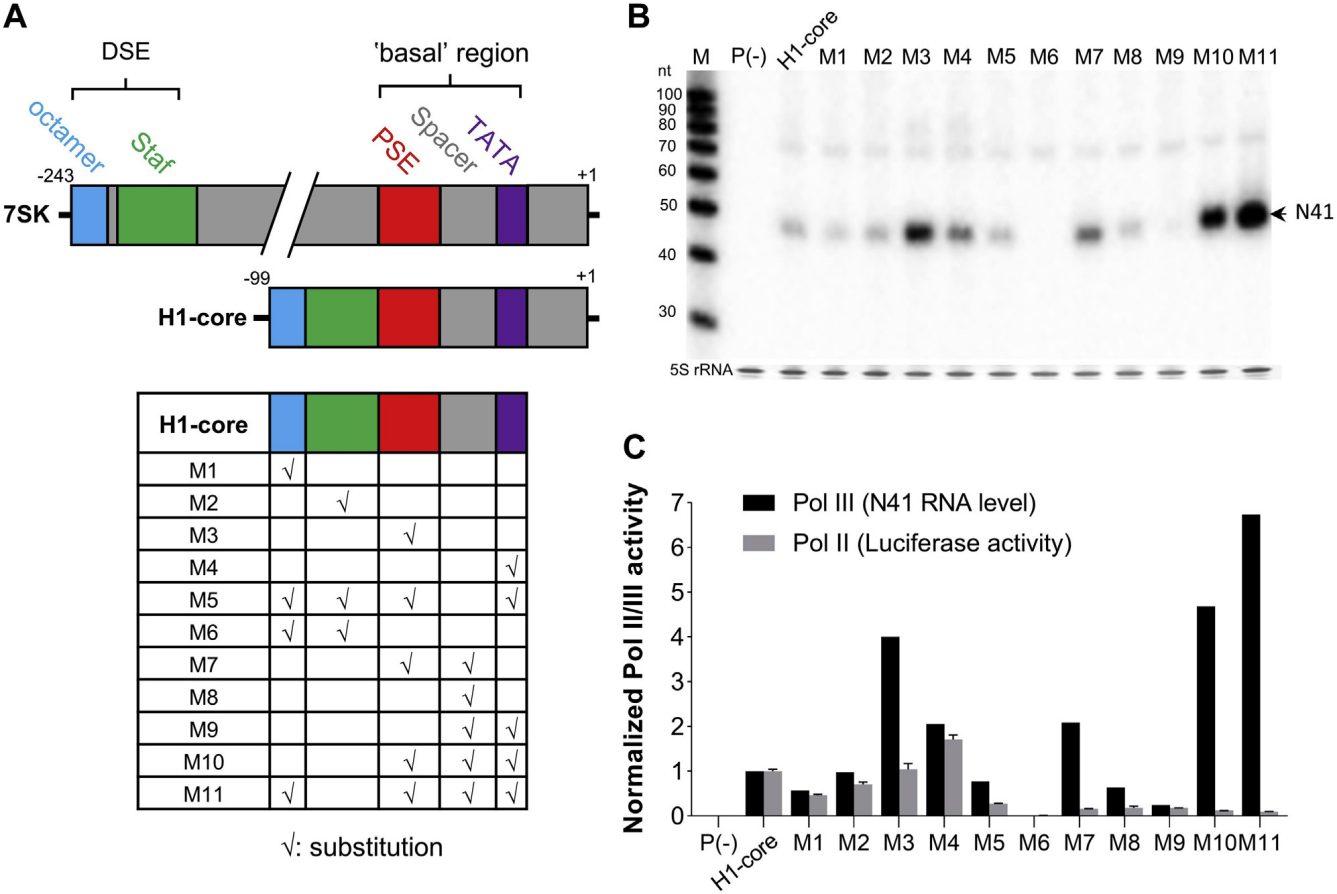
**Introducing 7SK elements in the H1-core promoter.***A,* schematic of the 7SK and H1-core promoters with the elements marked in colors. The color code was used in the table insert to mark the 7SK elements introduced in the H1-core to generate the M1-11 variants. *B,* N41 transcript detection by Northern blotting as described for Figure 1*B*. The promoter-less P(−) construct was used as a negative control. M depicts the RNA size marker (nt). A representative blot of two experiments is shown. *C,* the Pol II/III activity is plotted for H1-core and the M1-11 variants. Pol III activity of the respective promoters was quantified based on N41 expression measured in (*A*). Luciferase activity reflecting Pol II activity was measured and quantified as described for Figure 1*C*. The data represent the mean ± S.D. (n = 3). The Pol II and III activity of H1-core was arbitrarily set at 1. DSE, distal sequence element; Pol II/III, RNA polymerase II/III; Staf, Staf binding site.

The H1-core promoter is particularly attractive for applications using viral vector systems that prefer a small Pol III expression cassette. However, its promiscuous Pol II activity is usually unwanted and can be problematic in the presence of a downstream RNA effector function (e.g., miRNA) or an open reading frame that is translated into an unwanted and possibly immunogenic protein. We therefore set out to engineer a Pol III-specific H1-core promoter. There is architectural similarity between the H1 and 7SK promoters (*upper panel* of Fig. 2*A*), but the latter lacks prominent Pol II activity. We reasoned that swapping of promoter elements from 7SK into H1 may transfer selective Pol III specificity onto the H1-core promoter.

We thus generated H1-core mutants by introducing individual or multiple 7SK elements (octamer, Staf, PSE, TATA box, and the spacer between the latter two motifs) and subsequently analyzed the Pol II/III activity profile. We transfected these 11 mutant constructs (M1-11) into HEK293T cells and measured the Pol II and III activity by luciferase assays and Northern blotting, respectively. The mutants varied profoundly in N41 expression, which reflects the Pol III strength, compared with the parental H1-core promoter (Fig. 2*B*, quantitation is shown in Fig. 2*C*). We identified three hybrid constructs with significantly increased Pol III activity (M3, M10, and M11). M3, in which the PSE was replaced, showed a 4-fold increase, indicating that the 7SK PSE supports Pol III transcription to a greater extent than the H1 PSE. Intriguingly, this substitution did not affect the Pol II activity, suggesting that Pol II to III competition is not the underlying mechanism for the increased Pol III activity. Constructs M10 and M11 carry multiple 7SK elements (three and four, respectively) and enhance the Pol III activity to an even greater extent than M3 (4.5- and 6.5-fold, respectively).

Pol II activity of the mutant constructs also varied considerably and did not seem to strictly follow the Pol III activity profile (Fig. 2*C*). Most mutants displayed reduced Pol II activity compared with H1-core, except for M3 that exhibited similar activity and M4 that showed increased activity. The most robust reduction in Pol II activity was apparent on combinatorial replacement of multiple elements (M5-7 and M9-11), with M11 showing the most dramatic reduction (11-fold), followed by M10 (8.5-fold). The M11 construct is the most Pol III-specific promoter, exhibiting the highest Pol III activity combined with a profoundly reduced Pol II activity. We note that M11, in which multiple H1 elements (octamer, PSE, spacer, and TATA box) were replaced, has more than 50% of its sequence derived from 7SK. It may thus be appropriate to call M11 a hybrid H1/7SK promoter, although this is a semantic issue. M11 characteristics are summarized in Figure 5.

### Assessing the Pol III-specific M11 promoter in different cell types

**Figure 3:**
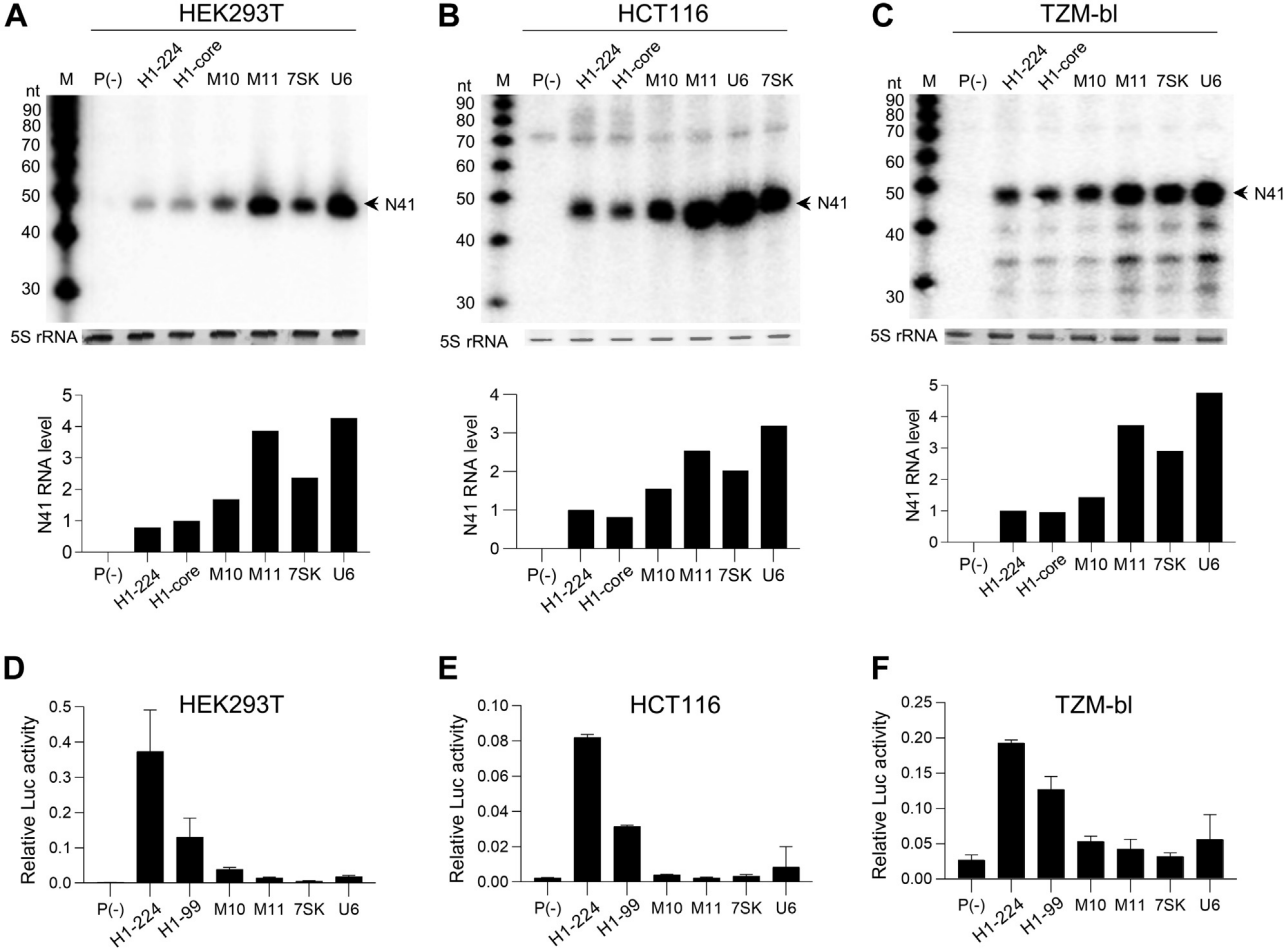
**The Pol III/II activity of M11 is cell type independent.** Northern blotting for the short N41 transcript was conducted for transfected HEK293T (*A*), HCT116 (*B*), and TZM-bl (*C*) cells as described for Figure 1*B*. Representative blots of at least two experiments are shown per cell type. Luciferase activity was measured for transfected HEK293T (*D*), HCT116 (*E*), and TZM-bl (*F*) cells as described for Figure 1*B*. The data represent the mean ± S.D. (n = 3). HCT116, colon cancer cell lines; HEK293T, human embryonic kidney 293T cells; TZM-bl, JC53-bl (clone 13).

In HEK293T cells, M11 is the promoter of choice for Pol III applications because of its high Pol III activity and minimal Pol II activity. To allow applications in different fields, it is important that this hybrid promoter functions similarly in different cell types, each with a distinct pool of transcription factors. To test this, we compared the Pol III activity of M11 with H1-224, H1-core, M10, and two popular Pol III promoters (7SK and U6) in three cell lines of various origins. The DNA constructs were transfected into HEK293T, colon cancer cell lines (HCT116), and JC53-bl [clone 13] (TZM-b1) cells, and RNA was extracted after 2 days for Northern blot analysis (Fig. 3, *A*–*C*, quantification is shown in *lower panels* of each graph). We observed a very similar Pol III activity profile in these cell types: the H1-224 and H1-core promoters have similar Pol III activity, which is boosted in M10 and especially in M11. Perhaps remarkable, the M11 promoter is more active than the two parental H1-core and 7SK promoters.

We next performed luciferase assays to verify that the low Pol II activity of M10 and M11 is a general feature of these promoters. Luciferase activity was measured 2 days after DNA transfection into HEK293T (Fig. 3*D*), HCT116 (Fig. 3*E*), and TZM-bl cells (Fig. 3*F*). A similar activity profile was observed in all cell types. H1-224 and H1-core have high Pol II activity, which is significantly reduced for M10 and especially M11. Note that M11 harbors similarly low Pol II activity as the 7SK promoter (5). We conclude that the enhanced Pol III activity and specificity of the novel H1/7SK hybrid promoter M11 does not seem to depend on the cell lineage.

### Exploring the use of H1 as Pol II-specific promoter

**Figure 4:**
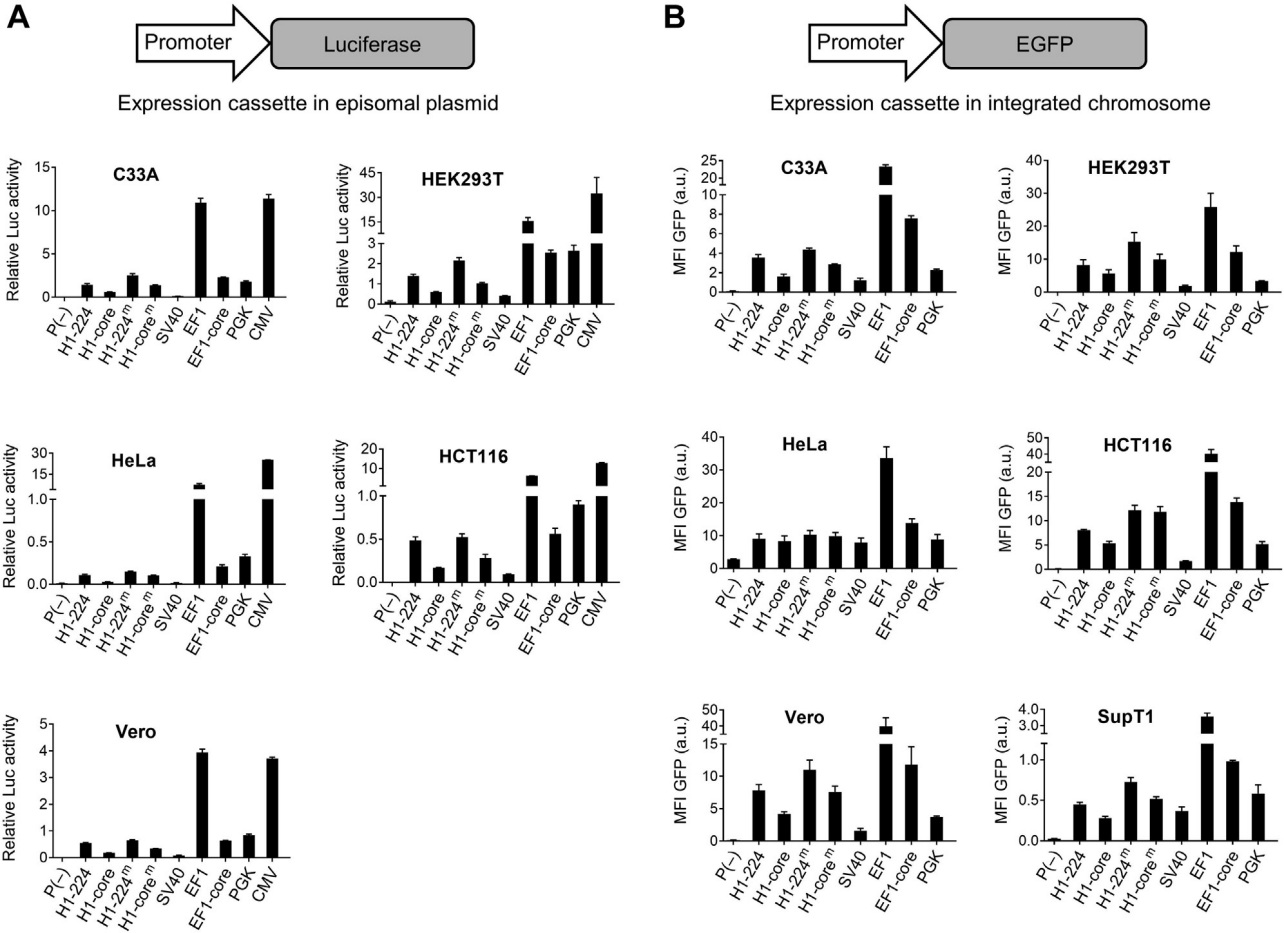
**The Pol II activity of H1 promoter variants *versus* classical Pol II promoters in multiple cell types.***A,* luciferase activity obtained for the indicated promoters in episomal plasmids in five cell types. Plasmids containing promoter-luciferase cassettes were subjected to a dual-luciferase assay, which was performed as described for Figure 1*C*. *B,* the mean GFP fluorescence in six cell types transduced with lentiviral vectors with the GFP reporter and driven by the indicated promoters. All cell types were transduced at the same MOI (0.25). After 5 days, the cells were analyzed by flow cytometry, and the mean fluorescence intensity (MFI) was determined. The data represent the mean (±S.D.) of three biological replicates. EF1, elongation factor 1; HCT116, colon cancer cell lines; HEK293T, human embryonic kidney 293T cells.

Because of the unusual and robust Pol II activity of the small H1 promoter, we decided to study its potential as promoter of choice for mRNA/protein production. Construct H1-224 is clearly the most Pol II active, but the H1-core promoter, which is less than half the size, also shows decent activity. In thinking about further improvement of the Pol II activity, we reasoned that inactivation of the TATA box would not only knockout Pol III activity but also boost Pol II activity as we reported for H1-230 (5). We therefore generated the TATA box mutants H1-224^m^ and H1-core^m^ and tested the Pol II activity. We transiently transfected the set of H1 promoter-luciferase constructs in five cell types (C33A, HEK293T, HeLa, HCT116, and Vero) and measured luciferase activity after 2 days (Fig. 4*A*). The luciferase signal varied widely across the cell types, but a clear general trend was apparent for the H1 variants. We observed that H1-224 is roughly 2-fold more active than H1-core, in line with the results obtained in HEK293T cells (Fig. 1*C*). Both H1-224^m^ and H1-core^m^ showed enhanced Pol II activity compared with the respective parental promoters in most of the tested cell lines, albeit to various extents. Mutation of the TATA box enhanced Pol II activity to a greater extent in H1-core^m^ than in H1-224^m^, but H1-core^m^ remained weaker than H1-224 and H1-224^m^. Thus, H1-224^m^ is the H1 variant with the highest Pol II activity, whereas H1-core^m^ is the promoter of choice when the promoter size forms a restriction.

To get a better grasp of the absolute Pol II activity, we compared the four H1 variants to five classical Pol II promoters (SV40 early, cytomegalovirus [CMV], elongation factor 1 [EF1], EF1-core, and phosphoglycerate kinase [PGK]). The size of these promoters is listed in Table 1, underscoring the significant size advantage of H1-core^m^. The EF1 and CMV promoters direct the highest luciferase expression level across the cell lines and are also the largest. EF1-core is 5- to 35-fold less active than the full-length EF1 promoter but exhibits similar Pol II activity as PGK. SV40 early is the weakest promoter. Remarkably, the small H1-224^m^ promoter is comparable in Pol II activity to EF1-core and PGK, thereby confirming that H1-224^m^ is a *bona fide* Pol II promoter that sustains high level Pol II transcription.

**Table 1:** Lengths and sources of the promoters used in this study

Promoter	Length (bp)	Sources
H1-224	224	This study
H1-core	99	This study
H1-224^m^	224	This study
H1-core^m^	99	This study
SV40 early	197	Addgene plasmid #E1761
EF1a	1179	(16)
EF1a-core	212	Addgene plasmid #52962
CMV	584	(6)
PGK	488	(17)

SV40, simian virus 40.

**Figure 5:**
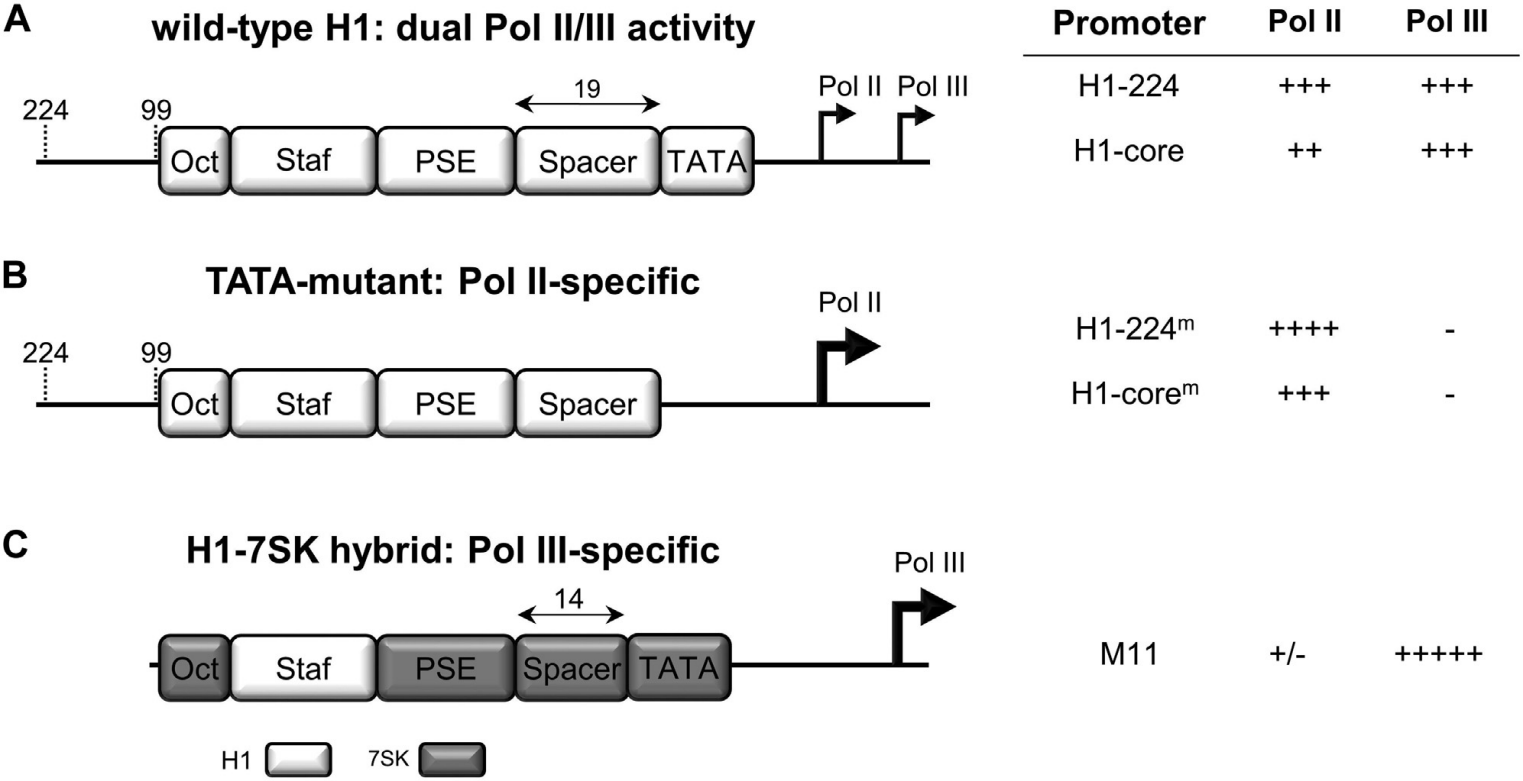
**Promoter characteristics of H1 variants.***A,* wild-type H1 and H1-core promoters harbor both Pol II and Pol III activity. *B,* deletion of the TATA box (TATA to TCGA) renders the H1 promoter Pol II specific and increases Pol II activity. H1-224^m^ was reported to lack Pol III activity (5) and deduced that H1-99^m^ has the same phenotype because all relevant promoter elements are located in the H1-99 region. Note that the spacer function is lost as a consequence of TATA-box deletion. *C,* replacement of the octamer, PSE, spacer, and TATA box of the H1 promoter by 7SK sequences yields a pol III-specific H1/7SK hybrid promoter with increased Pol III activity. Pol II/III, RNA polymerase II/III; PSE, proximal sequence element; Staf, Staf binding site.

As durable gene therapy applications will likely use transgene cassettes that become stably integrated into the human genome, we next assessed the activity of the novel H1 promoters in the chromosomal context (Fig. 4*B*). Lentiviral vectors containing promoter-GFP expression cassettes were constructed and transduced into six cell lines. The CMV construct was excluded because the same sequences are already present in the lentiviral vector backbone, which may cause unwanted recombination events during lentiviral vector production and subsequent transduction (8). We used a low multiplicity of infection of 0.25 for lentiviral transduction to ensure a single integration event per cell. The mean GFP fluorescence intensity (mean fluorescence intensity) was measured by flow cytometry at 5 days after transduction. The results in Fig. 4*B* are largely consistent with the luciferase data (Fig. 4*A*), indicating that H1-224^m^ is the most active H1 variant across the cell lines tested. All four H1 variants exhibit greater activity than the SV40 promoter and are comparable in strength to the EF1-core and PGK promoters. In conclusion, the novel H1-224^m^ and H1-core^m^ promoters exhibit robust Pol II activity that is maintained in a chromosomal context, arguing that both promoters could become the promoter of choice for Pol II applications. H1-224^m^ offers the advantage of potency, but H1-core^m^ would be useful when the size of the transgene cassette is a concern, *e.g.*, in viral vectors. H1-224^m^ and H1-core^m^ characteristics are summarized in Fig. 5.

## Discussion

The H1 promoter is a type 3 RNA Pol III promoter and is popular for the expression of small noncoding RNAs (1, 2). It was recently disclosed that type 3 Pol III promoters can also sustain a certain level of Pol II transcription and that the H1 promoter has unusually high Pol II activity (5). In this study, we report that the 99-bp H1-core promoter is the minimal segment required for both Pol II and Pol III activity. Pol III activity of H1-core is equal to that of the full-length promoter, whereas Pol II activity is somewhat reduced.

The dual Pol II/III activity of the H1 promoter is an undesired feature in many applications. For example, in gene silencing applications where the H1 promoter is routinely used to express short shRNA transcripts, the silencing efficacy could be compromised as both Pol II and Pol III transcripts will fold the same hairpin structure that actually can refold to form an extended intermolecular base-pairing interaction between the two transcripts. In this way, the extended Pol II transcript can interfere with the folding and function of the short Pol III shRNA transcript. In addition, extended Pol II transcription initiated from the H1 promoter may run into downstream Pol II transcription units and cause head-to-head collision of the polymerase complexes or transcriptional interference (9, 10). Finally, if an open reading frame is located downstream of the H1 promoter, the unwanted Pol II transcript can be translated into a protein with potentially toxic or immunogenic properties, a scenario that should especially be avoided in a gene therapy setting, where an immune response can trigger the specific killing of the transgene-containing cells (11, 12, 13). Such potential drawbacks can become more serious when Pol III promoters are multiplexed for certain applications (4, 8).

As dual promoter activity is generally undesired, we aimed to generate Pol II-and Pol III-specific H1 variants. Engineering of a Pol III-specific H1 promoter was achieved by replacing H1 promoter elements by those of 7SK, a popular type 3 Pol III promoter with high Pol III specificity (5). Replacement of the basal region (containing PSE, spacer, and TATA box) together with the 7SK octamer yielded the H1/7SK hybrid promoter M11 with minimal Pol II activity and increased Pol III activity compared with both parental promoters. The M11 promoter is a valuable supplement to the current Pol III toolbox that we recommend for pure Pol III applications instead of H1 or U6. Another application of M11 relates to multiplexing of gene cassettes, in which each cassette has its own promoter. The use of identical promoters is not advised as sequence repeats are prone to recombination, especially in viral vectors (8). Expression cassettes with different promoters should be used instead (8). For instance, the novel Pol III-specific M11 promoter could be combined with 7SK as the sequence homology is limited.

Our results also provide insight into why the complete basal domain of 7SK is required for maximal Pol III activity and specificity. PSE-containing promoters come in two flavors: with or without a TATA box (14). Promoters with both a PSE and TATA box generally sustain Pol III transcription, whereas PSE-only promoters support Pol II transcription. In Pol II preinitiation complexes (PICs), the snRNA-activating protein complex binds the PSE and recruits both the TATA-box binding protein and the transcription factors TFIIA/TFIIB that subsequently promote Pol II recruitment. snRNA-activating protein complex and TATA-box binding protein are also recruited to promoters with a PSE and TATA box, but TFIIB-related factor 1 or 2 (b-related factor 1/2 [BRF1/BRF2]) is assembled into the PIC instead of TFIIA and TFIIB. PICs with BRF1/2 then selectively recruit Pol III. Thus, Pol III PICs are bound to both the PSE and TATA box and bridge the distance between these elements, which constitutes the spacer (Fig. 5). The spacer length is therefore critical and ranges from 15 to 19 bps (15). Indeed, insertion or deletion of just 5 bp inactivates the U6 Pol III promoter (16) and illustrates that the PSE element, spacer, and TATA box are major determinants of Pol II/III specificity.

Chromatin immunoprecipitation sequencing experiments demonstrated that PSE-containing promoters without TATA almost exclusively recruit Pol II, whereas those with a TATA box recruit Pol III (15). The H1 promoter is truly exceptional in that it recruits both Pol II and III. Therefore, the real question is why only the H1 promoter allows both Pol II and Pol III transcription. When compared with 13 other PSE/TATA promoters, two H1 features stand out: H1 has the shortest TATA box (together with U6-7 and U6-8) and H1 has the longest spacer. Taken all findings together, a scenario emerges in which the spacer in the H1 promoter is simply too big for efficient bridging of the PSE and TATA box (Fig. 5). If the PIC is only bound to the PSE, but not the TATA box, Pol II expression is likely favored, similar to PSE-only promoters. We thus hypothesize that the spacer is a critical determinant of Pol III specificity. In line with this hypothesis is that all 7SK spacer-containing mutants (M7-M11) harbor very low Pol II activity.

Unexplained remains the low Pol III activity of M8 and especially M9, in which the spacer or spacer and TATA box were replaced by 7SK elements, where one may have expected higher Pol III activity. Close inspection of the relevant sequences provides a possible solution. The H1 PSE that we replaced is TCACCATAAACGTGAAAT (7, 15), whereas the consensus PSE of U6/7SK is STSACCGTGWST(GT)RAAR(0-3)TG (17, 18). The H1 PSE element seems to lack the 3'-terminal G nucleotide, but the base directly downstream of the H1 PSE is in fact a G. This raised some doubt whether the defined H1 PSE sequence is correct. To test this, we mutated this 3'-terminal G into T and scored strongly reduced Pol II and Pol III activity (unpublished results). This indicates that mutants M8 and M9, which maintain the RAAR(0-3)TG consensus PSE sequence, do carry a 2-bp insertion (underlined in TCACCATAAACGTGAAATTTG), which may explain their reduced Pol III activity.

Some of the results may also suggest that the mechanism of Pol II/III specificity is even more complex. For example, in mutant M3, we have replaced the H1 PSE by that of 7SK and kept the H1 spacer length and TATA box. This mutant exhibits four times increased Pol III specificity over the parental H1 promoter by specifically increasing Pol III activity. Thus, the 7SK PSE element intrinsically sustains higher Pol III activity and specificity compared with the H1 PSE. Mutant M4 in which we replaced the H1 TATA box is also noteworthy as the 7SK TATA box enhances promoter activity, although not specificity, which may also contribute to the enhanced Pol III activity of M10 and M11.

In conclusion, we propose that the complete basal 7SK region is required for maximal Pol III activity/specificity for at least three reasons: (1) the intrinsic high Pol III activity/specificity of the 7SK PSE; (2) the optimal 7SK spacer length for PIC binding to the PSE and TATA box; and (3) the strong 7SK TATA box that generally enhances Pol II and III activity.

To engineer Pol II-specific H1 promoters, we mutated the TATA box of two H1 size variants, H1-core and H1-224, and termed these promoters H1-core^m^ and H1-224^m^. We previously used this TATA trick to render the H1 promoter strictly Pol II specific (5). Both H1 variants possess Pol II activity that is similar to that of the commonly used Pol II promoters EF1-core and PGK. H1-core^m^ is slightly less active than H1-224^m^. But if the size of the gene construct is an important factor, the 99 bp H1-core^m^ promoter forms an attractive alternative.

The Pol II-specific H1 promoter may have some specific advantages over regular Pol II promoters for gene expression purposes. First, the Pol II-specific H1-224^m^ variant is only 224 bp in length, which equals the size of EF1-core, but is much smaller than many commonly used Pol II promoters (Table 1). H1-core^m^ with a size of 99 bp is even smaller and among the smallest available Pol II promoters, including synthetic minipromoters such as the super core promoter family (19). The limited size of these two H1 promoter variants may provide a distinct advantage in settings where a transgene is delivered by viral vectors with a limited packaging capacity. This problem is for instance encountered in lentiviral vectors that express the Cas9 endonuclease and a second transgene. Because of the large size of the *Cas9* gene, small promoters such as EF1 core are frequently used for its expression. If a second transgene has to be expressed, one can use different molecular biology strategies (internal ribosome entry site [IRES], 2A), but this usually results in a greatly reduced expression of the second transgene. It may thus be beneficial that the second transgene has its own promoter. When such size restrictions are apparent, a small and highly active Pol II promoter like H1-224^m^ or H1-core^m^ is highly recommended.

Second, our initial tests indicate that the Pol II promoter activity of H1-224^m^ and H1-core^m^ is independent of the cell lineage. This may have been expected as the physiological role of the H1 promoter is to drive the expression of the RNA component of RNase P, the enzyme involved in tRNA maturation in all cell types (20). In other words, the Pol III activity of the natural H1 promoter is essential in all cells, but we currently do not know the function of the Pol II activity. Third, we demonstrated that the Pol II-specific H1-224^m^ promoter is comparable in strength to the commonly used EF1-core and PGK promoters and that H1-core^m^ is only a bit weaker. Both promoters thus possess sufficiently high Pol II activity to be considered as general Pol II promoters. Fourth, we selectively knocked out the Pol III activity of H1-224^m^ and H1-core^m^, whereas some of the regularly used Pol II promoters may exhibit unwanted Pol III activity. Although such activity has not been reported, it may have escaped our attention as small transcripts were simply not analyzed. Similar to the disadvantage of unwanted expression of extended RNA transcripts by Pol III promoters, one can imagine that short RNA expression by Pol II promoters may cause unwanted side effects.

Finally, various cell types like hematopoietic and stem cells are prone to transcriptional or promoter silencing, and the use of the strong viral CMV promoter is not recommended for this very reason. H1-based Pol II-specific promoters are of particular interest as their Pol III origin may make them less prone to silencing than canonical Pol II promoters. This would make biological sense for the H1 promoter as there is a continuous requirement for the H1 transcript of the RNase P ribonucleoprotein complex. Thus, especially for gene therapy applications, it would be important to test the longevity of H1-224^m^- and H1-core^m^–mediated gene expression. In conclusion, this work adds new small Pol II- and Pol III-specific promoters to the molecular biology toolbox with some distinct properties.

## Experimental procedures

### Constructs

To engineer the H1-N41-luciferase construct, we ordered a gBlock gene fragment (Integrated DNA Technologies) encoding full-length H1-374 promoter-N41 sequence. This DNA fragment was ligated into the pGL3-basic vector using NheI and NcoI restriction enzyme sites by Gibson cloning, which constitutes the backbone construct pGL3-H1-374. To generate H1 constructs with 50-bp serial promoter truncations, the H1-truncation variants were PCR amplified and then ligated into pGL3-H1-374 on the NheI and XhoI sites. To generate H1-mutant constructs, gBlock gene fragments encoding the H1-mutant sequences (Figs. S1 and S2) were ordered and ligated into pGL3-H1-374 on the NheI and XhoI sites by Gibson cloning according to the protocol (New England Biolabs). We made five constructs in which luciferase is driven by a classical Pol II promoter: SV40 early promoter, human elongation factor 1 alpha (EF1a) promoter, human EF1a core promoter (EF1a-core), CMV immediate-early promoter, and the mouse PGK-1 promoter. The promoter lengths and sequences are summarized in Table 1 and Supplementary Data S1, respectively. The gBlock promoter fragments were ordered and ligated into pGL3-H1-374. All constructs were verified by Sanger sequencing using the BigDyeTerminator v1.1 Cycle Sequencing Kit (Applied Biosystems).

### Cell culture

HEK293T, C33A, HCT116, HeLa, TZM-bl, and Vero cells were grown as monolayer in DMEM (Invitrogen) supplemented with 10% fetal calf serum (FCS), penicillin (100 U/ml), streptomycin (100 U/ml), and minimal essential medium nonessential amino acids at 37 °C and 5% carbon dioxide. HCT116 is a human colon cancer cell line and TZM-bl is a HeLa cell derivative that was engineered by amphotropic retroviral transduction to express CD4, CCR5, and CXCR4. SupT1 cells were grown in advanced RPMI (Gibco BRL, Carlsbad, CA) supplemented with L-glutamine, 1% FCS, penicillin (30 U/ml), and streptomycin (30 μg/ml) at 37 °C and 5% carbon dioxide. Cells were trypsinized and seeded 1 day before transfection. For Northern blotting experiments, 1 × 10^6^ cells in 2 ml medium were seeded per well in 6-well plate. For luciferase reporter assays, 1.5 × 10^5^ cells in 0.5 ml medium were seeded per well in 24-well plate.

### Northern blotting

Northern blotting for N41 detection was performed as described previously. Briefly, equimolar amounts of the DNA constructs (equivalent of 3 ug pGL3-H1-374 plasmid) were transfected using Lipofectamine 2000 (Invitrogen). Total cellular RNA was extracted 2 days post-transfection using the mirVana miRNA isolation kit (Ambion), and 5 μg of total RNA with loading buffer was preheated for 5 min at 95 °C and then resolved in a 15% denaturing polyacrylamide gel (Precast Novex TBU gel, Life Technologies). The γ[^32^P]-labeled decade RNA marker (Life Technologies) was used for size estimation. To check for equal sample loading, the gel was stained in 2 mg/ml of ethidium bromide for 20 min and visualized under UV light. The RNA in the gel was transferred to a positively charged nylon membrane (Boehringer Mannheim). Locked nucleic acid oligonucleotides (Pol47: 5'-ATTACTACTGCCCCTTCAC-3') were 5'-end labeled with [^32^P]-ATP (0.37 MBq/ml; PerkinElmer) using kinaseMax kit (Ambion). The unincorporated nucleotides were removed by Sephadex G-25 spin columns (Amersham Biosciences). The membrane was incubated overnight with purified locked nucleic acid oligonucleotides in 10 ml ULTRAhyb hybridization buffer at 42 °C. The membrane was washed with low (2× saline sodium citrate and 0.1% SDS) and high (0.1× saline sodium citrate and 0.1% SDS) stringency buffers. The signals were captured by Typhoon FLA 9500 (GE Healthcare Life Sciences) and quantitated using ImageJ software.

### Dual-luciferase reporter assay

Equimolar amounts of H1-based constructs (equivalent of 200 ng empty pGL3-H1-374) and 2 ng of Renilla luciferase plasmid were cotransfected into cells in 24-well plates using Lipofectamine 2000 (Invitrogen) according to the manufacturer's instructions. At 48 h post-transfection, luciferase activity was measured with the dual-luciferase reporter assay kit (Promega, Madison, WI), according to the manufacturer's protocol. The ratio of firefly to Renilla luciferase was calculated to represent the relative firefly luciferase activity. Three independent transfections were performed.

### Lentivirus production and transduction

Lentivirus was produced similarly as described previously (6). Briefly, HEK293T cells were seeded in T75 square-centimeter flasks 1 day before transfection to achieve a confluency of approximately 80% at the day of transduction. The same molar amounts of GFP lentiviral constructs were transfected together with a fixed amount of packaging plasmids pSYNGP, pRSV-rev, and pVSV-g, using X-tremeGENE (Roche). The medium was replaced 18 h after transfection by 8 ml of OptiMEM (Invitrogen). The culture supernatant was collected at 24 h post-transfection, and transduction of SupT1 T cells was performed by serial dilution to assess the titer of the lentivector stock. For measuring GFP expression from integrated chromosome, a multiplicity of infection of 0.25 was used for transduction of HEK293T, C33A, HCT116, HeLa, Vero, and SupT1 cells.

### Flow cytometry

Promoter activity from an integrated chromosomal position was measured 5 days after transduction with lentiviral vectors that express GFP, which was monitored by flow cytometry. Harvested cells were washed with fluorescence-activated cell sorting buffer (PBS with 2% FCS), and data acquisition was performed by an LSR Fortessa (BD Biosciences) interfaced with the fluorescence-activated cell sorting-Diva software system. The mean fluorescence intensity of GFP was analyzed by FlowJo version X software (FlowJo, LLC).

## Data availability

All representative data are contained within the article.

## Conflict of interest

The authors declare that they have no conflicts of interest with the contents of this article.
